# Congenital bronchopleural fistula in a newborn: a rare cause of persistent neonatal air leak – a case report

**DOI:** 10.3389/fped.2026.1932384

**Published:** 2026-08-19

**Authors:** Tomasz Janowicz, Michał Szostawicki

**Affiliations:** Clinical Department of Pediatric Surgery and Urology School of Public Health, University of Warmia and Mazury in Olsztyn, Olsztyn, Poland

**Keywords:** congenital airway malformation, congenital bronchopleural fistula, neonatal pneumothorax, persistent air leak, preterm neonate, thoracoscopy, thoracotomy

## Abstract

**Background:**

Congenital bronchopleural fistula is an exceptionally rare cause of persistent neonatal air leakage. Most bronchopleural fistulas in newborns are related to mechanical ventilation, infection, or iatrogenic injury. The clinical presentation can suggest pneumothorax commonly seen in premature infants, diagnosis is often delayed, and an ongoing uncontrolled air leak may significantly compromise effective ventilation.

**Case presentation:**

A female neonate was delivered at 29 weeks of gestation with a birth weight of 1,360 g and Apgar scores of 6, 7, and 9. Immediately after birth, she developed progressive respiratory failure. Bilateral pneumothoraces were diagnosed, including a left tension pneumothorax. Despite two pleural drains and active suction, a continuous air leak persisted at approximately 800 mL/min, corresponding to the infant’s entire minute ventilation. Chest computed tomography demonstrated a communication between the left bronchial tree and the pleural cavity, consistent with a bronchopleural fistula. Dedicated CT angiography was not performed because of deterioration and the need to avoid delaying definitive treatment. Selective bronchial intubation, temporary bronchial occlusion with a Fogarty catheter, and minimally invasive options were considered. Owing to respiratory instability despite drainage, surgical treatment was selected. Thoracoscopy identified the lesion, but secure closure could not be achieved thoracoscopically; the procedure was converted to thoracotomy, and the fistula was excised with a segment of adjacent lung tissue. Postoperative bronchoscopy revealed tracheobronchial stenosis and left main bronchial malacia, which were managed conservatively. Genetic testing identified PAI-1/SERPINE1 and MTHFR variants, but these did not establish a recognised syndrome or causal association with the fistula. The postoperative respiratory course was favourable. At 18 months of age, the child had no recurrent pneumothorax, chronic respiratory symptoms, or late surgical complications.

**Conclusion:**

Persistent high volume air leakage despite adequate pleural drainage should lead to suspect a major bronchial lesion and investigate congenital pulmonary and airway malformations. Conservative and bronchoscopic strategies may be effective in selected stable patients, whereas early surgery may provide definitive control in rapidly deteriorating neonates with a localised structural lesion. This case highlights the diagnostic value of CT, the need for individualised treatment selection, and the possibility of sustained long-term recovery after surgical repair.

## Introduction

A bronchopleural fistula is an abnormal connection between a bronchus and the pleural cavity that causes persistent air leakage (1). In neonates, most reported fistulas are acquired and related to iatrogenic trauma, mechanical ventilation, infection, or other injury to the tracheobronchial tree (1, 2). Congenital bronchopleural fistula is exceptionally rare and has been described mainly in isolated case reports. Its prevalence in the neonatal population is unknown (1, 2).

Diagnosis is challenging because the initial presentation may resemble pneumothorax associated with prematurity or mechanical ventilation. Persistent high-volume air leakage despite correctly positioned pleural drains should indicate the possibility of a bronchopleural fistula or major bronchial injury (1, 2). Reported treatments include pleural drainage, ventilatory modification, selective bronchial intubation, temporary bronchial occlusion, endobronchial closure, surgery, and, in selected cases, extracorporeal membrane oxygenation (ECMO) (1–9).

Congenital bronchopleural fistula should also be differentiated from congenital pulmonary airway malformation, bronchopulmonary sequestration, hybrid lung malformations, congenital bronchial atresia, bronchogenic cysts, and communicating bronchopulmonary foregut malformations (10–13). These conditions differ anatomically but may cause severe neonatal respiratory disorder and require detailed imaging.

## Aim of the study

The aim of this report is to describe the diagnosis and treatment of a very preterm infant with a presumed congenital left bronchopleural fistula, with emphasis on persistent air leakage, differential diagnosis, and individualised treatment selection.

## Case study

A female neonate was delivered vaginally at 29 weeks of gestation, with a birth weight of 1,360 g and Apgar scores of 6, 7, and 9. This was the mother's third pregnancy and third delivery. The two previous pregnancies had ended in premature deliveries at 24 and 28 weeks, and both newborns had died, according to the mother, in circumstances similar to those observed in the present patient. Medical records, imaging, genetic results, and post-mortem findings from those pregnancies were unavailable; therefore, the similarity of the presentations could not be verified.

Immediately after birth, the neonate was transferred to the neonatal intensive care unit because of rapidly progressive respiratory failure and grade III respiratory distress syndrome. Bilateral pneumothoraces were diagnosed, including a left-sided tension pneumothorax.

**Figure 1:**
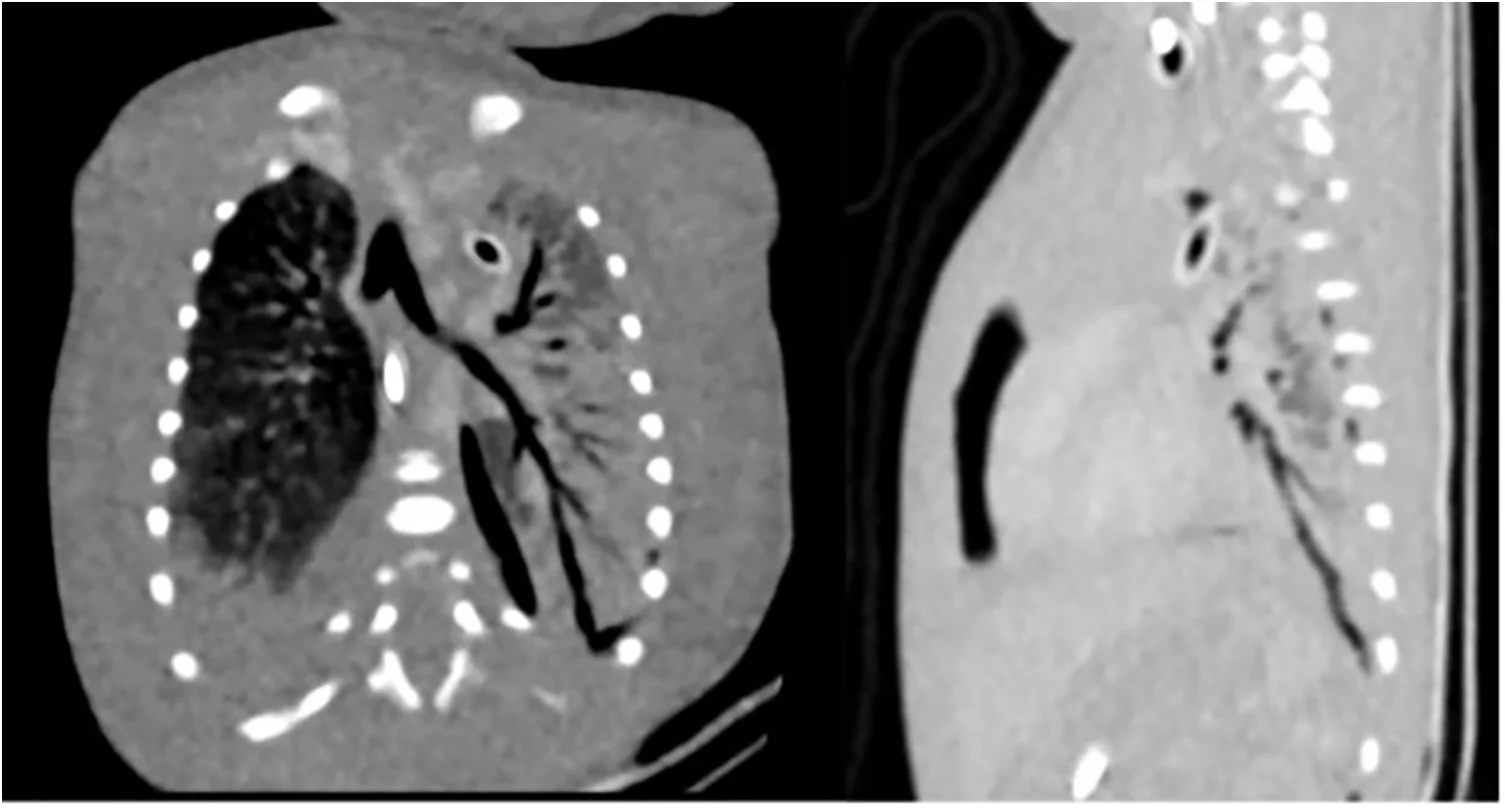
CT scan taken before the procedure (bronchopleural fistula is indicated by the arrow).

The pleural cavity was decompressed. Despite two pleural drains and active suction up to 20 mm H₂O, a continuous air leak persisted, reaching approximately 800 mL/min and corresponding to almost the infant's entire minute ventilation (Figure 1). Oxygen desaturation continued, and respiratory stabilisation could not be achieved.

**Figure 2:**
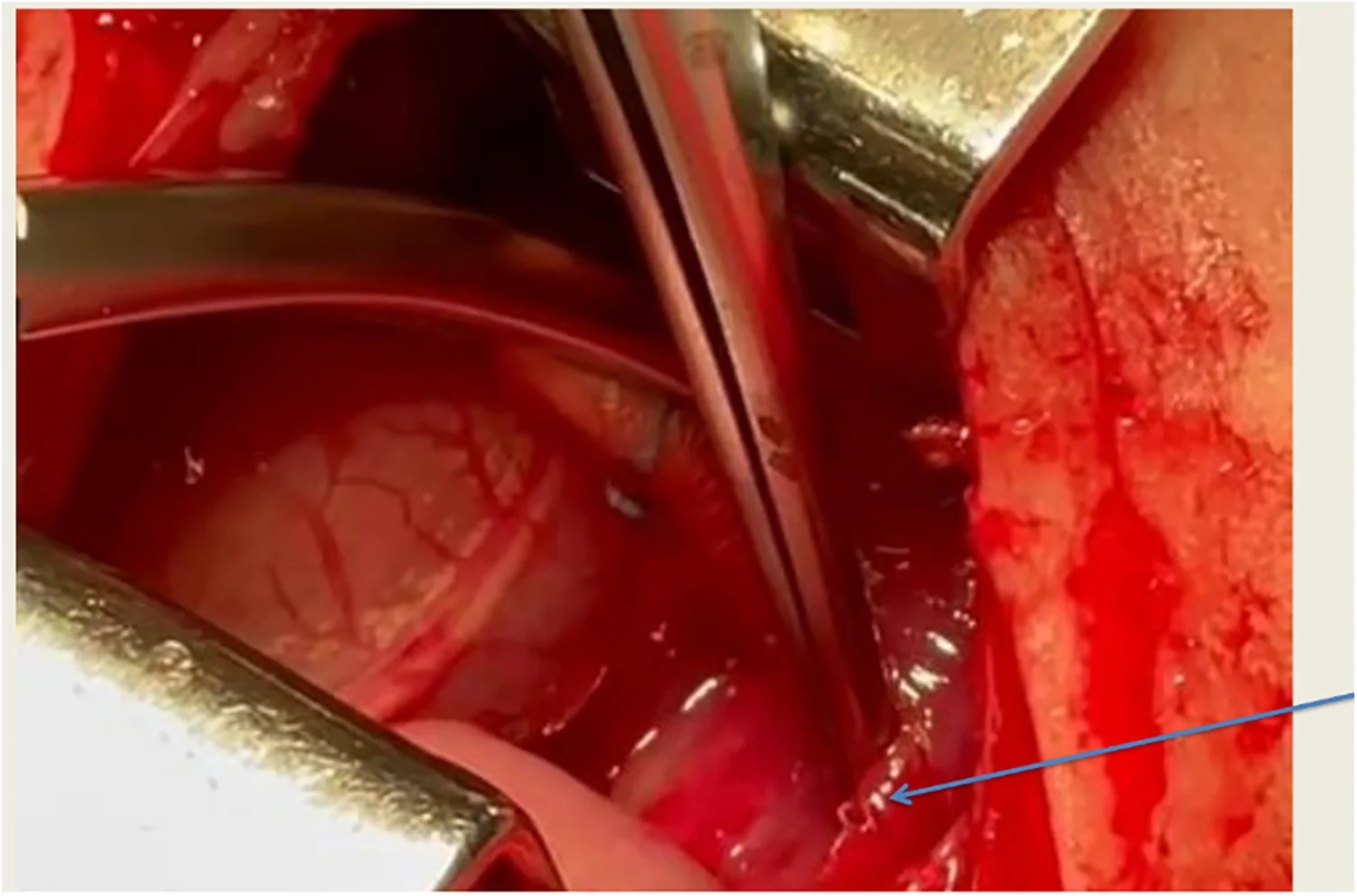
Intraoperative view of bronchopleural fistula (arrow).

A congenital pulmonary or bronchial defect was suspected. Chest CT demonstrated a communication between the left bronchial tree and the pleural cavity consistent with a bronchopleural fistula (Figure 2). Dedicated CT angiography was not performed because the patient's condition was rapidly deteriorating and definitive treatment could not be delayed. The available CT localised the suspected fistula; however, the absence of a dedicated vascular phase limited complete preoperative exclusion of pulmonary sequestration, a hybrid lung malformation, or another congenital pulmonary vascular anomaly.

**Figure 3:**
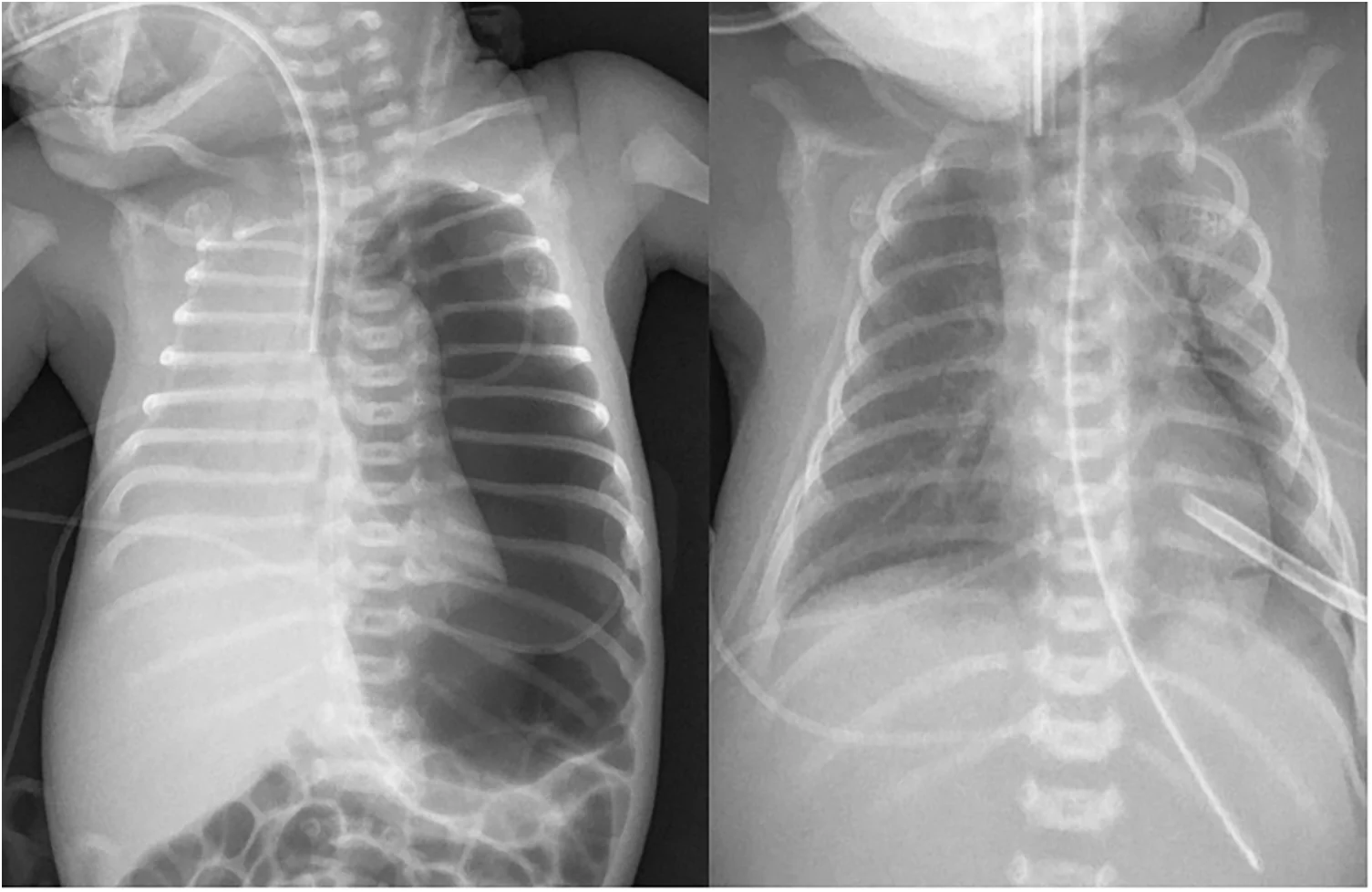
X-ray taken before and after drainage of the pleural cavity.

The neonate was referred for urgent surgery. Thoracoscopy identified the lesion, but secure definitive closure could not be achieved thoracoscopically. The procedure was converted to thoracotomy, and the fistula was excised with a small segment of adjacent lung tissue (Figure 3).

Selective bronchial intubation, temporary occlusion with a Fogarty catheter, and other minimally invasive options were considered. However, because of the rapidly deteriorating clinical condition and failure to achieve respiratory stabilisation despite pleural drainage, urgent surgical treatment was considered the most appropriate option.

Postoperative bronchoscopy demonstrated mild stenosis of the trachea and both main bronchi near the bifurcation and left main bronchial malacia. These findings did not require urgent intervention and were managed conservatively.

Further treatment concerned prematurity and its complications. Grade III/IV intraventricular haemorrhage, retinopathy of prematurity, a patent foramen ovale, and a haemodynamically insignificant patent ductus arteriosus were diagnosed. Progressive atrophy of the right kidney was also identified. Ultrasonography showed a 16-mm right kidney with poorly defined corticomedullary differentiation, heterogeneous echogenicity, trace peripheral vascularisation, and cysts up to 2 mm.

Genetic testing identified a heterozygous PAI-1/SERPINE1 variant and a homozygous MTHFR variant. The available report did not provide sufficient information regarding exact nomenclature, pathogenicity, or functional consequences. These findings did not establish a recognised genetic syndrome or a causal relationship with the fistula.

**Figure 4:**
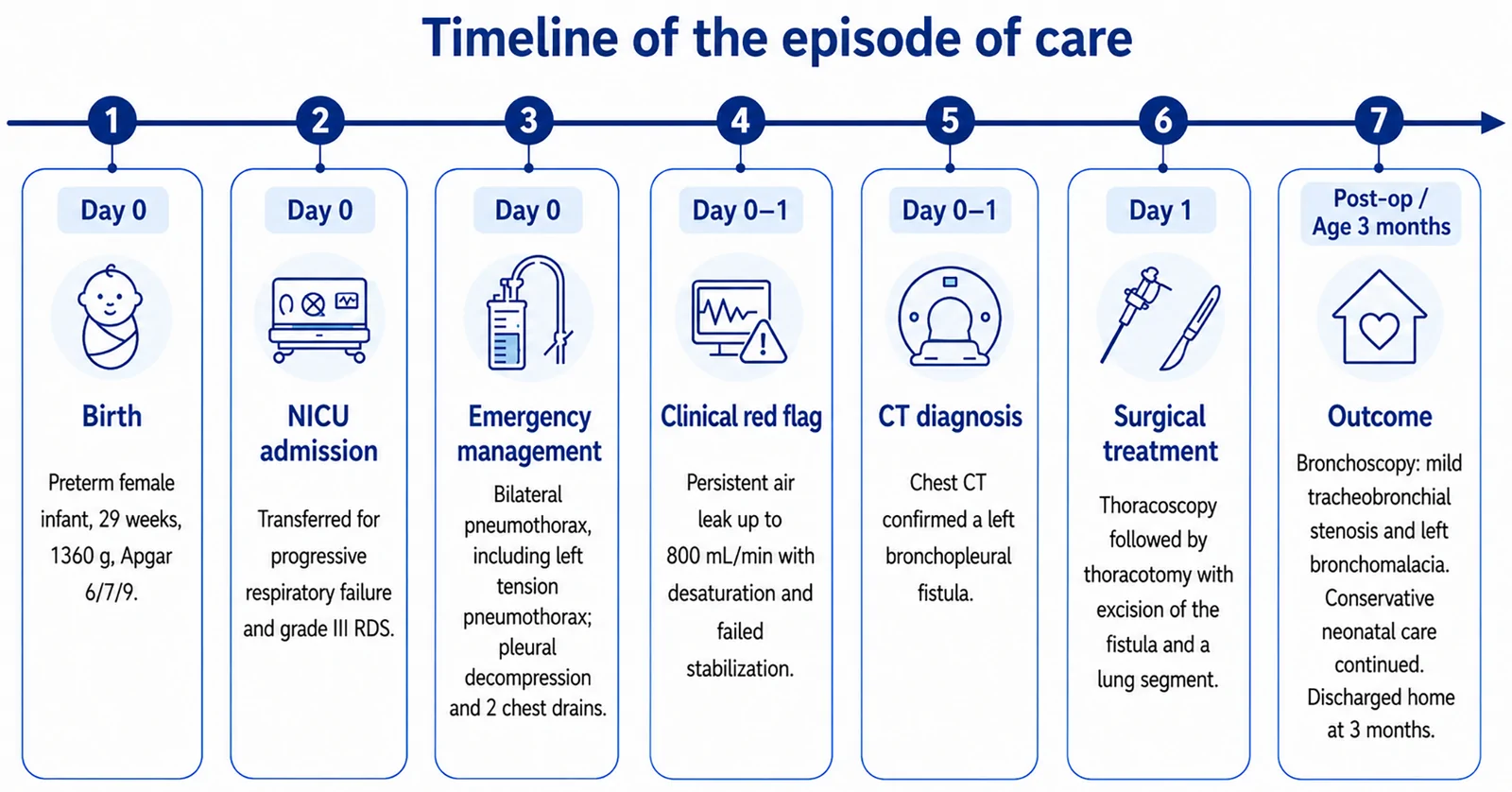
Timeline of the episode of care.

The postoperative respiratory course was favourable. No recurrent pneumothorax or clinically significant persistent air leak occurred. The infant was discharged home at 3 months of chronological age (Figure 4).

The patient remained under regular follow-up in a neonatal outpatient clinic. At 18 months of chronological age, growth and development followed the expected course for a child born prematurely. No recurrent pneumothorax, chronic respiratory symptoms, or late complications related to the fistula or its surgical treatment were observed.

## Discussion

Congenital bronchopleural fistula in a neonate is exceptionally rare. Most neonatal bronchopleural fistulas reported in the literature are acquired and associated with inflammation, trauma, airway instrumentation, or mechanical ventilation (1, 2). In the present case, the immediate onset of respiratory compromise, persistent high volume leakage, CT findings, and intraoperative identification of the lesion supported a presumed congenital origin, although this could not be confirmed by a specific histopathological or molecular marker.

Persistent air leakage despite adequate pleural drainage should raise suspicion of a bronchopleural fistula or major bronchial injury (1, 2). In this patient, the leak approached the entire minute ventilation and prevented effective respiratory stabilisation. This differed from a small or progressively decreasing air leak that might be managed with continued drainage and lung protective ventilation.

The differential diagnosis includes other congenital pulmonary and airway malformations. Communicating bronchopulmonary foregut malformations involve an abnormal connection between the respiratory system or dysplastic lung tissue and the oesophagus or stomach (11). No such communication was demonstrated clinically, radiologically, bronchoscopically, or intraoperatively.

Hybrid lung malformations combine features of congenital pulmonary airway malformation and bronchopulmonary sequestration and may include cystic or dysplastic lung tissue supplied by an aberrant systemic artery (12). Pulmonary sequestration consists of non-functioning pulmonary tissue with a systemic arterial supply and usually no normal connection with the tracheobronchial tree. Contrast-enhanced CT or CT angiography can demonstrate feeding vessels and venous drainage (12, 13). In the present patient, CT angiography was not performed because additional imaging would have delayed urgent treatment. Therefore, sequestration or a hybrid lesion could not be excluded preoperatively with complete radiological certainty.

Congenital bronchial atresia is characterised by interruption of a proximal bronchus, a blind-ending distal bronchus, bronchocele formation, air trapping, and hyperinflation. Recent literature emphasises the importance of CT in differentiating this anomaly from infection and other structural lesions (10). In this case, imaging and surgery demonstrated a bronchopleural communication rather than an interrupted bronchus, making bronchial atresia unlikely.

The mild tracheobronchial stenosis and left bronchial malacia may indicate a broader developmental airway abnormality. However, these findings do not define a recognised syndrome or establish a direct embryological link with the fistula.

No standard treatment algorithm exists. Management should be based on clinical stability, leak volume, fistula location, and available neonatal, bronchoscopic, and surgical expertise. Conservative treatment may include pleural drainage, reduced ventilatory pressures, HF ventilation, positioning, permissive hypercapnia, and observation. In a published group of 18 neonates with persistent air leakage suggestive of bronchopleural fistula, most improved with drainage and suction without selective bronchial intubation or surgery (1). Thus, surgery is not universally required.

Selective bronchial intubation can reduce airflow through the fistula while maintaining ventilation of the contralateral lung, and successful neonatal use has been reported (3). Temporary bronchial occlusion with a Fogarty catheter is another rescue option. In an extremely preterm infant, intermittent occlusion with a 3-Fr Fogarty catheter combined with high-frequency oscillatory ventilation produced clinical and radiological improvement (4). These methods require reliable localisation of the affected bronchus and sufficient stability for airway manipulation.

Other reported rescue strategies include endobronchial tissue adhesives and ECMO-supported lung-rest ventilation (7, 8). These approaches may be effective in selected cases but depend on lesion anatomy, institutional resources, and patient stability. ECMO is also associated with substantial haemorrhagic, thrombotic, neurological, and infectious risks in very premature infants.

In the reported case, conservative and minimally invasive options were considered. However, the rapidly deteriorating condition, persistent high volume air leak, and failure to achieve stabilisation despite pleural drainage led to the decision for urgent surgery.

Surgery remains appropriate when conservative or bronchoscopic measures are ineffective, anatomically unsuitable, unavailable, or unlikely to provide timely control. Published reports describe successful surgical repair in very-low-birth-weight and very preterm infants after failure of drainage, selective ventilation, or pleurodesis (2, 5, 6). In the present case, thoracoscopy allowed localisation, while thoracotomy provided adequate exposure for secure excision.

The available literature directly addressing presumed congenital bronchopleural fistula in very preterm neonates is extremely rare. Most reports concern acquired bronchial injury, ventilator-associated fistula, or persistent pneumothorax without a clearly demonstrated congenital lesion. Therefore, the present case supports individualised therapeutic approach.

The family history of two previous premature neonatal deaths with reportedly similar respiratory failure raises concern regarding a possible recurrent familial, congenital, or genetic predisposition. However, the absence of documentation prevents confirmation that the siblings had the same lesion. Prematurity and respiratory failure are non-specific and may have had different causes.

The coexistence of bronchopleural fistula, mild tracheobronchial stenosis and malacia, and a structural right renal abnormality may descriptively suggest multiple congenital anomalies involving more than one organ system. Nevertheless, these findings do not correspond to a recognised malformation syndrome.

The PAI-1/SERPINE1 and MTHFR variants were reported because the family history and associated abnormalities prompted a search for a possible common genetic background. No comparable cases linking these variants with congenital bronchopleural fistula or a reproducible bronchial-renal phenotype were identified. PAI-1 polymorphisms have mainly been studied in relation to fibrinolysis, thrombosis, pregnancy loss, and selected neonatal respiratory conditions (14). Common MTHFR polymorphisms have limited clinical value in thrombophilia assessment and do not define a congenital malformation syndrome (15). The variants should therefore be regarded as findings of uncertain or possibly incidental significance.

## Limitations

This single-patient report cannot establish the superiority of surgical treatment over conservative, bronchoscopic, or extracorporeal strategies. The presumed congenital origin was based on immediate presentation, imaging, and intraoperative findings but could not be confirmed by a specific histopathological or molecular marker.

Dedicated CT angiography was not performed because of the rapidly deteriorating condition and need for urgent treatment. Consequently, pulmonary sequestration, a hybrid lung malformation, or another vascular anomaly could not be excluded preoperatively with complete radiological certainty.

The history of the siblings was based on maternal report and could not be verified. The exact nomenclature and pathogenic classification of the PAI-1/SERPINE1 and MTHFR variants were unavailable.

No directly comparable published case was identified. Specifically, no previous report was found describing a presumed congenital bronchopleural fistula in a very preterm neonate presenting immediately after birth with a massive air leak and requiring urgent thoracoscopy followed by thoracotomy and fistula excision. This limits comparison with previously reported diagnostic and therapeutic experience.

Although follow-up to 18 months showed no recurrent pneumothorax, chronic respiratory symptoms, or late surgical complications, longer observation and formal pulmonary function assessment will be required to evaluate outcomes later in childhood.

## Conclusions

This case describes an exceptionally rare presumed congenital bronchopleural fistula in a 29-week preterm infant presenting immediately after birth with a massive air leak resistant to pleural drainage. The case demonstrates a diagnostic and therapeutic pathway from recognition of an air leak approaching the entire minute ventilation, through CT and thoracoscopic localisation, to definitive surgical excision.

Persistent high-volume air leakage despite correctly positioned drains should prompt early consideration of a major bronchial lesion and a broader differential diagnosis of congenital pulmonary and airway malformations. CT is essential for localisation and treatment planning. CT angiography should be considered when vascular congenital pulmonary malformations are suspected, provided that the patient's condition permits further imaging.

Conservative treatment, selective bronchial ventilation, Fogarty-catheter occlusion, and other endobronchial techniques may be effective in selected stable patients. Surgery is not universally required. However, in a rapidly deteriorating neonate with an uncontrollable air leak and a localised structural lesion, early surgical intervention may provide definitive control.

The favourable postoperative course and absence of recurrent pneumothorax, chronic respiratory symptoms, or late surgical complications at 18 months indicate sustained respiratory recovery. The associated congenital abnormalities and genetic variants do not currently establish a recognised syndrome or explain the fistula.

## Data Availability

The original contributions presented in the study are included in the article/Supplementary Material, further inquiries can be directed to the corresponding author.
